# From mitochondria to tumor suppression: ACAT1's crucial role in gastric cancer

**DOI:** 10.3389/fimmu.2024.1449525

**Published:** 2024-08-23

**Authors:** Wei He, Yanfang Li, Song-Bai Liu, Ying Chang, Shiyuan Han, Xingyu Han, Zixin Ma, Hesham M. Amin, Yao-Hua Song, Jin Zhou

**Affiliations:** ^1^ Cyrus Tang Hematology Center, Soochow University, Suzhou, China; ^2^ Suzhou Key Laboratory of Medical Biotechnology, Suzhou Vocational Health College, Suzhou, China; ^3^ Department of Hematopathology, the University of Texas MD Anderson Cancer Center, Houston, TX, United States; ^4^ Department of General Surgery, the First Affiliated Hospital of Soochow University, Suzhou, China

**Keywords:** gastric cancer, ACAT1, tumor stem cells, EMT, mitochondrial enzyme

## Abstract

Acetyl CoA acetyltransferase 1 (ACAT1), a mitochondrial enzyme, is mainly involved in the formation and decomposition of ketones, isoleucine, and fatty acids. Previous clinical studies showed that mutations in the *ACAT1* gene lead to ketoacidosis, Notably the role of *ACAT1* in human cancer’ pathogenesis varies depending on cancer type, and its specific role in gastric cancer remains largely unknown. In the current study, we found that the expression of ACAT1 in primary late-stage gastric cancer tumor tissues was significantly lower than in early-stage tumors. This observation was further confirmed in high-grade gastric cancer cell line MKN45. The expression of CD44 and OCT4 was decreased, while CD24 expression was increased by overexpressing *ACAT1* in MKN45 gastric cancer cells. Moreover, the ability of gastric cancer cells to form colonies on soft agar was also reduced by *ACAT1* overexpression. Likewise, overexpression of *ACAT1* inhibited epithelial mesenchymal transition (EMT) in gastric cancer cells evidenced by increased expression of the epithelial marker E-Cadherin, decreased expression of mesenchymal marker vimentin, and decreased expression levels of SNAI 1/3. In addition, *ACAT1* overexpression inhibited cell migration and invasion, improved the response to 5-Fluorouracil (5-FU) and etoposide. In contrast, inhibition of ACAT1 activity promoted the proliferation of gastric cancer cells. The xenotransplantation results in nude mice showed that overexpression of *ACAT1* in gastric cancer cells inhibited tumor growth *in vivo*. In addition, the low expression of *ACAT1* in gastric cancer was further validated by searching public databases and conducting bioinformatic analyses. Mechanistically, bioinformatic analysis found that the inhibitory effect of *ACAT1* in gastric cancer may be related to the Adipocytokine Signaling Pathway, Ppar Signaling Pathway, Propanoate Metabolism and P53 Signaling Pathway. Correlation analysis indicated *ACAT1* mRNA expression was correlated with immune infiltrates. Collectively, our data show that ACAT1 induces pronounced inhibitory effects on gastric cancer initiation and development, which may impact future strategies to treat this aggressive cancer.

## Introduction

Cancer stem cells (CSCs) possess inherent ability of self-renewal and tumor-initiating. CSCs are pluripotent as they can produce diverse cell types that differentiate into different tissues that make up the entire tumor (1). To survive in hostile microenvironment and evade the host’s immune system, cancer cells undergo changes in gene expression, transition between cell types, and alter metabolism. These adaptations are crucial in for cancer establishment, growth, metastasis, and treatment resistance. CD44 and OCT4 have emerged as survival biomarkers in various solid tumors, including gastric cancer (2). CD44 expression is linked to promoting cancer cell proliferation, self-renewal, and metastasis (3–6). OCT4 contributes to tumor transformation, drug resistance (7), and invasion (8–10). In gastric cancer stem cells, CD44 is overexpressed while CD24 is under expressed (11, 12).

Epithelial mesenchymal transition (EMT) is a dynamic process involving the transformation of epithelial cells into mesenchymal cells. For cancer cells to migrate from their original location and infiltrate blood vessels, they must undergo changes that enhance their flexibility by altering the cytoskeleton. These adaptations allow tumor cells to breach the walls of small blood vessels and enter the bloodstream (13). Once in the bloodstream, these cells can migrate to invade distant organs. During EMT, epithelial cells lose their adhesion molecules, which leads to the loss of apical-basal polarity (14). EMT is also characterized by a decrease in the expression of the epithelial cell marker E-cadherin, and an increase in the mesenchymal marker vimentin (15). Understanding the roles of cancer stem cells and EMT markers is essential for advancing cancer treatment strategies.

Acetyl CoA acetyltransferase 1 (ACAT1) was initially linked to β-ketothiolase deficiency, where mutations in *ACAT1* gene result in recurrent episodes of ketosis characterized by vomiting, dehydration, and shortness of breath (16–19). More recent studies have associated ACAT1 expression and cancer. Increased levels of ACAT1 have been observed in cancers of the prostate, endometrium, breast, and liver. Conversely, ACAT1 levels are decreased in renal cell carcinoma, nasopharyngeal carcinoma, and glioma. Elevated ACAT1 activity has also been detected in head and neck cancer, lung cancer, and leukemia. Notably, the expression and role of ACTA1 in gastric cancer is not completely understood. In this study, we investigated the correlation between ACAT1 expression and gastric cancer. Overexpression of *ACAT1* in gastric cancer cells resulted in decreased expression levels of CD44 and OCT4, while the expression level of CD24 increased. Additionally, gastric cells with overexpressed *ACAT1* exhibited significantly reduced migration and invasion abilities, and improved the response to chemotherapy drugs.

## Results

### The expression of ACAT1 is downregulated in advanced gastric cancer

**Figure 1:**
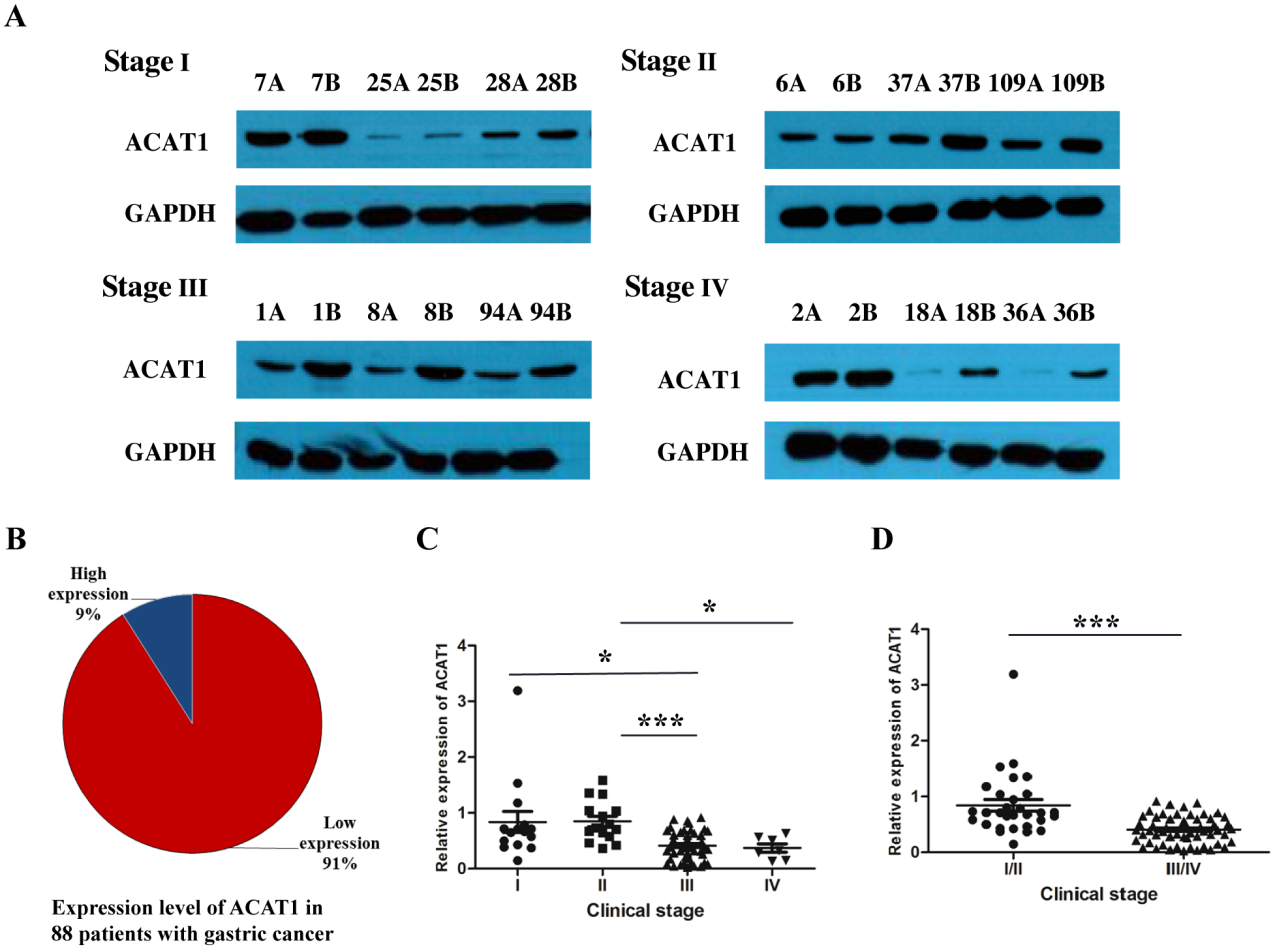
The relative expression of ACAT1 (cancer tissue/paracancerous tissue) was significantly decreased in patients with advanced gastric cancer. **(A)** Western blot analysis of protein expression of ACAT1 in gastric cancer patient tumor tissues at different stages. A: gastric cancer tissue, B: adjacent noncancerous area. **(B)** The proportion of patients with high expression of ACAT1 and those with low expression of ACAT1 in 88 pairs of samples with stage I-IV gastric cancer. **(C, D)** Relative expression of 88 pairs of gastric cancer patient samples. Relative expression = the normalized value of cancer tissue/the normalized value of adjacent tissue. Values are means ± SD. *p < 0.05, ***p < 0.001.

To analyze the expression of ACAT1 in gastric cancer, a Western blot analysis was performed on 88 pairs of gastric cancer and adjacent noncancerous tissue samples from patients at stages I-IV. The results showed that ACAT1 is expressed at significantly lower levels in gastric cancer tissues than in the adjacent benign tissues (**Figures 1A, B**). Notably, there was a marked reduction in ACAT1 expression in late-stage gastric cancer compared to early stage cancer tissues, suggesting that ACAT1 may have an inhibitory effect on the progression of gastric cancer (**Figures 1C, D**).

### ACAT1 exhibits the highest expression level in AGS cells

**Figure 2:**
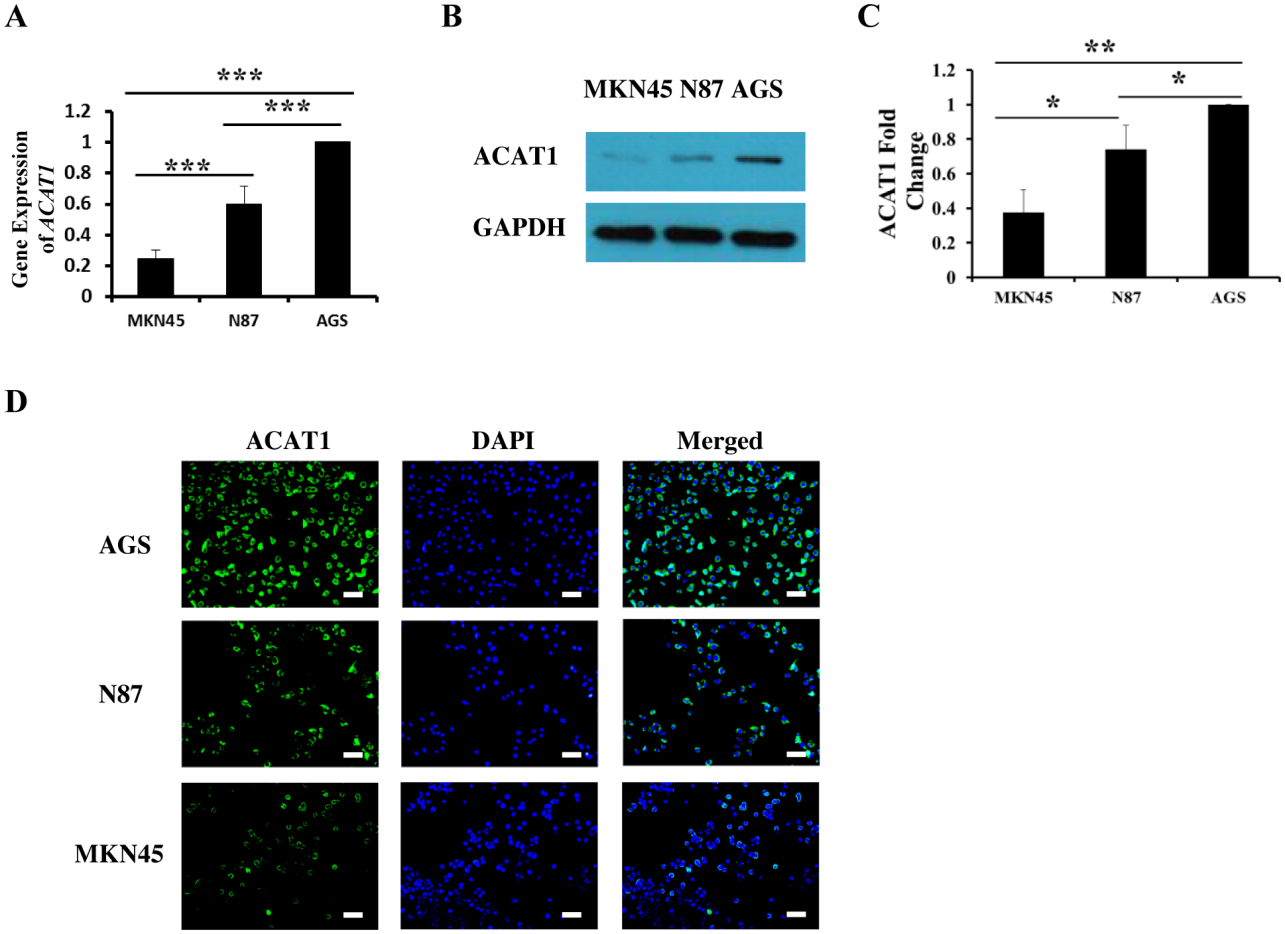
The expression of ACAT1 in gastric cancer cell lines MKN45, N87 and AGS. **(A)** RT-qPCR analysis of *ACAT1* mRNA. **(B, C)** Western blot analysis of ACAT1. **(D)** Immunofluorescence staining of ACAT1 (green). The nuclei were stained with DAPI (blue). Scale bar: 100 μm. n = 3 in each group. Values are means ± SD. *p < 0.05, **p < 0.01, ***p < 0.001.

To explore the function of ACAT1 in gastric cancer, we analyzed three different gastric cancer cell lines: MKN45, N87, and AGS. Using RT-qPCR and Western blot, we measured mRNA and protein expression levels of ACAT1 in these cell lines. Our data show that AGS cells have the highest expression of ACAT1, followed by N87 and then the MKN45 cells (**Figures 2A–C**). These expression levels were further confirmed through immunofluorescence staining (**Figure 2D**).

### ACAT1 inhibits the expression of CD44 and OCT4

**Figure 3:**
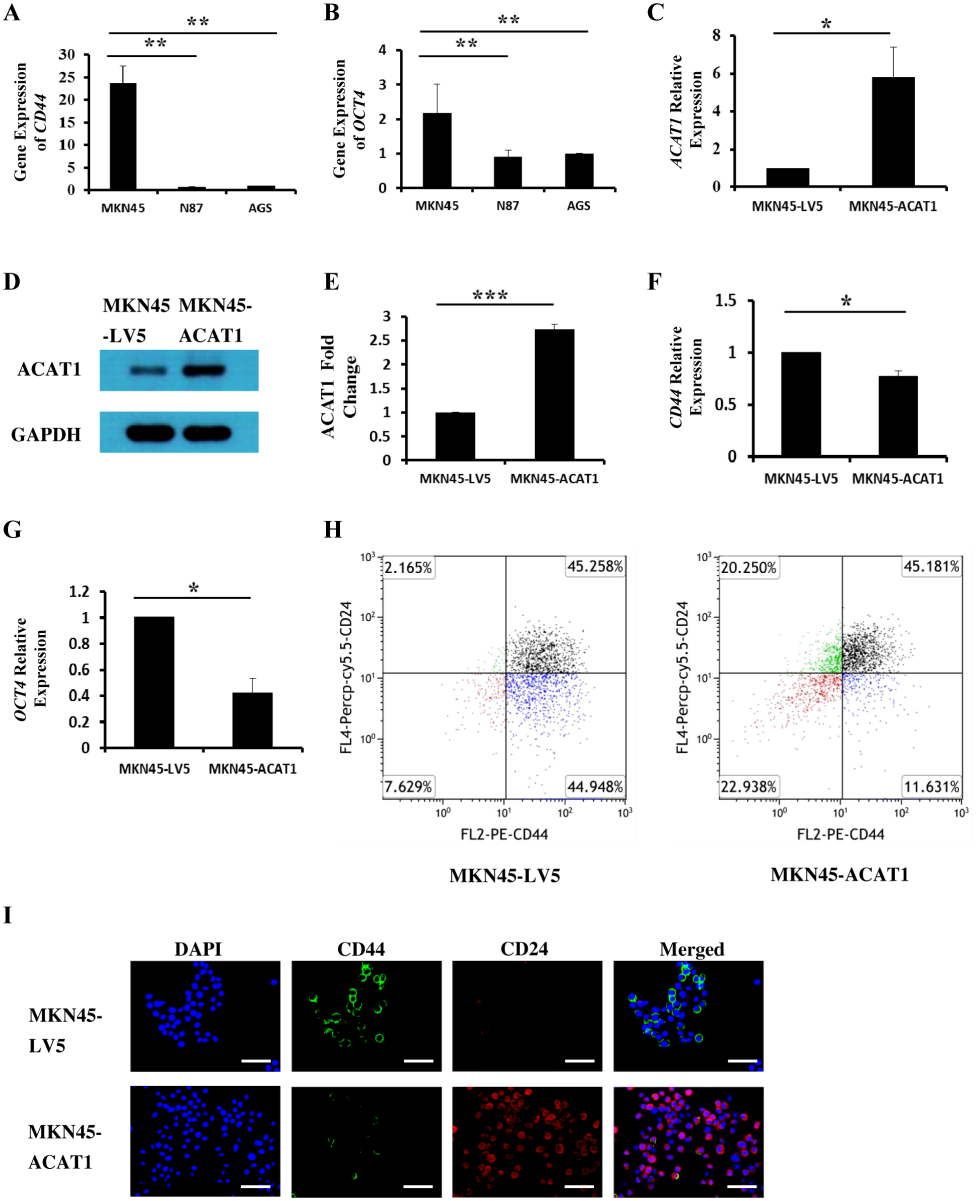
ACAT1 modulates the expression of stem cell markers. **(A, B)** RT-qPCR analysis of the mRNA expression of *CD44* and *OCT4* in gastric cancer cell lines MKN45, N87 and AGS. **(C–E)** RT-qPCR and Western blot to verify the overexpression of *ACAT1* in MKN45 cells. **(F, G)** RT-qPCR analysis of *CD44* and *OCT4* in MKN45-ACAT1 cells and MKN45-LV5 cells. **(H)** Flow cytometric analysis of CD44 and CD24 in MKN45-ACAT1 and MKN45-LV5 cells. **(I)** Immunofluorescence staining of CD44 (green) and CD24 (red) in MKN45-ACAT1 and MKN45-LV5 cells. The nuclei were stained with DAPI (blue). Scale bar: 100 μm. n = 3 in each group. Values are means ± SD. *p < 0.05, **p < 0.01, ***p < 0.001.

Cancer stem cells (CSCs) are a subset of cells capable of initiating tumor development. CD44 and OCT4 are commonly used indicators of gastric cancer stem cells (20, 21). We investigated the relationship between ACAT1 and the levels of CD44 and OCT4 in three different gastric cancer cell lines. The results show a significant decrease in the mRNA expression of *CD44* and *OCT4* in AGS and N87 cells compared to MKN45 cells (**Figures 3A, B**). These findings suggest a negative correlation between ACAT1 and gastric cancer stem cells markers expression, consistent with previous research (22) and our observation in patient samples.

To further investigate this possibility, we overexpressed *ACAT1* in MKN45 cells (**Figures 3C–E**) and examined its effect on the expression of tumor stem cell markers. RT-qPCR results demonstrated that, compared to MKN45-LV5 cells, overexpression of *ACAT1* significantly decreased mRNA expression of *CD44* and *OCT4* (**Figures 3F, G**).

According to existing literature, CD44 is highly expressed in stem cells from breast cancer (23), gastric cancer (11), and oral squamous cell carcinoma (24), while CD24 is typically expressed at lower levels. Our results show that overexpressing *ACAT1* in MKN45 cells led to a decrease in CD44-positive cells from 90.21% to 56.81%, and an increase in CD24-positive cells from 47.42% to 65.43%. Additionally, the proportion of cells with high CD44 and low CD24 expression was significantly reduced (**Figure 3H**). Immunofluorescence staining confirmed the presence of CD44 and CD24 proteins on the surface of gastric cancer cells, showing that increased ACAT1 levels suppress CD44 expression and enhance CD24 expression (**Figure 3I**). These findings provide further evidence that *ACAT1* reduces the expression of stem cell markers in gastric cancer cells.

### ACAT1 inhibits the EMT process by suppressing the expression of SNAI1 and SNAI3

**Figure 4:**
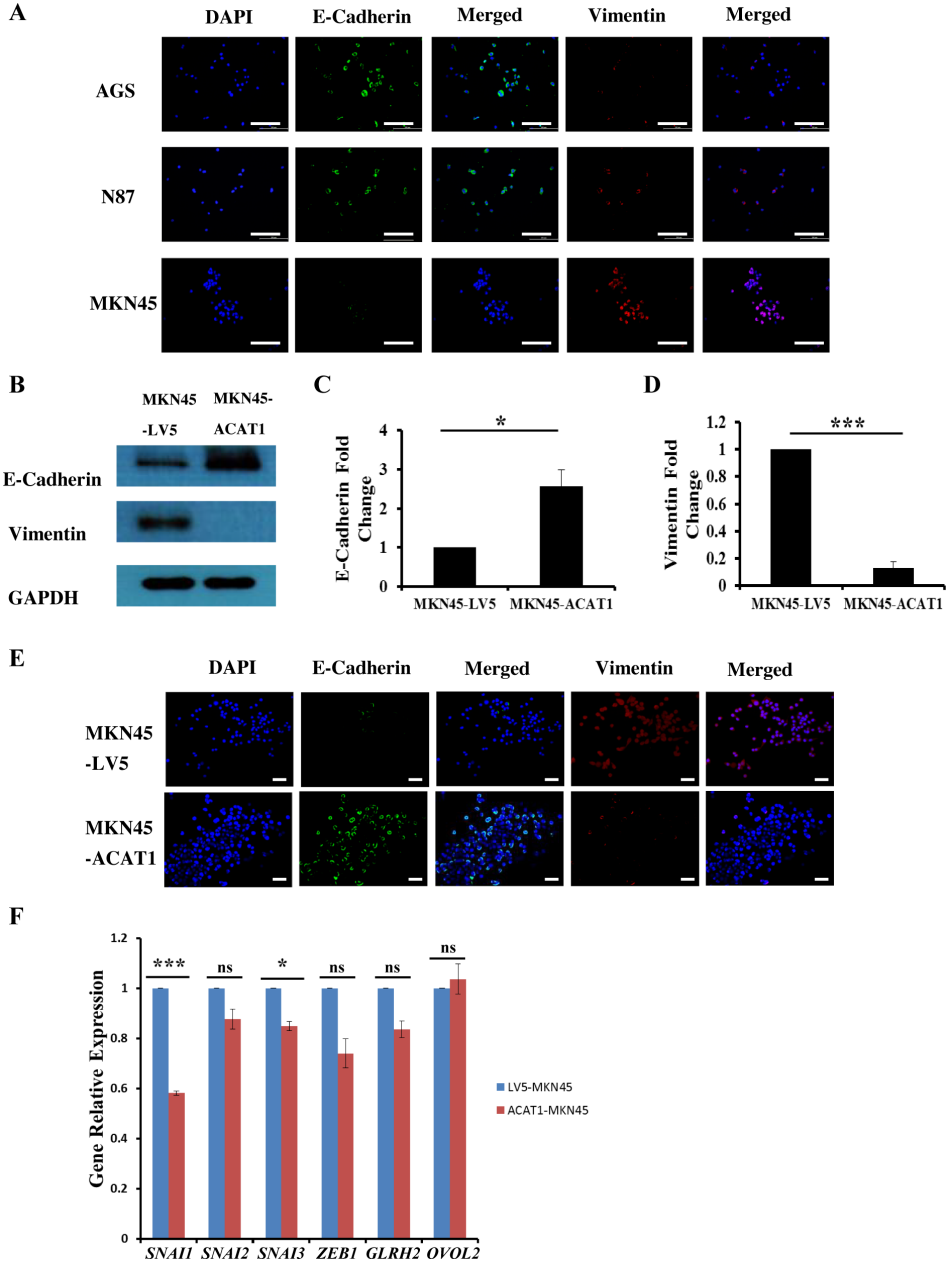
ACAT1 impedes the EMT process by inhibiting the expression of *SNAI1* and *SNAI3*. **(A)** Immunofluorescence staining of the EMT markers E-cadherin (green) and vimentin (red) in gastric cancer cell lines MKN45, N87 and AGS. The nuclei were stained with DAPI (blue). Scale bar: 200 μm. **(B–D)** Western blot was performed to analyze the levels of E-Cadherin and vimentin expression in MKN45-ACAT1 cells compared to MKN45-LV5 cells. **(E)** Immunofluorescence staining of E-Cadherin (green) and vimentin (red) in MKN45-ACAT1 and MKN45-LV5 cells. The nuclei were stained with DAPI (blue). Scale bar: 100 μm. **(F)** RT-qPCR analysis of EMT-related genes in MKN45-ACAT1 and MKN45-LV5 cells. n = 3 in each group. Values are mean ± SD. ns, not significant. *p < 0.05, ***p < 0.001.

Epithelial-mesenchymal transition (EMT) involves epithelial cells transforming into mesenchymal cells, taking on a fibroblast-like appearance (24, 25). EMT is characterized by a reduction in epithelial cell markers and an increase in mesenchymal cell markers, facilitating enhanced cell migration and invasion (26). Immunofluorescence staining was used to measure the levels of E-cadherin (an epithelial cell marker) and vimentin (a mesenchymal cell marker) in three gastric cancer cell lines. We found that E-cadherin expression is lowest in MKN45 cells and highest in AGS cells, while vimentin exhibits the opposite pattern (**Figure 4A**).

To further investigate the correlation between ACAT1 and EMT processes, we conducted experiments in MKN45 cells overexpressing *ACAT1*. Protein expression levels of EMT markers in MKN45-LV5 and MKN45-ACAT1 cells were assessed using Western blot analysis. Compared to MKN45-LV5 cells, overexpression of *ACAT1* in MKN45-ACAT1 cells enhanced E-cadherin and suppressed vimentin expressions (**Figures 4B–D**). These results were consistent when immunofluorescence staining was used (**Figure 4E**). To further analyze the role of ACAT1 in EMT, we measured the mRNA levels of EMT-related genes. The results showed a decrease in the expression of the EMT-promoting genes *SNAI1* and *SNAI3* when *ACAT1* was overexpressed (**Figure 4F**). This suggests that ACAT1 may impede the progression of EMT by suppressing these genes.

### ACAT1 inhibits the migration and invasion of gastric cancer cells

**Figure 5:**
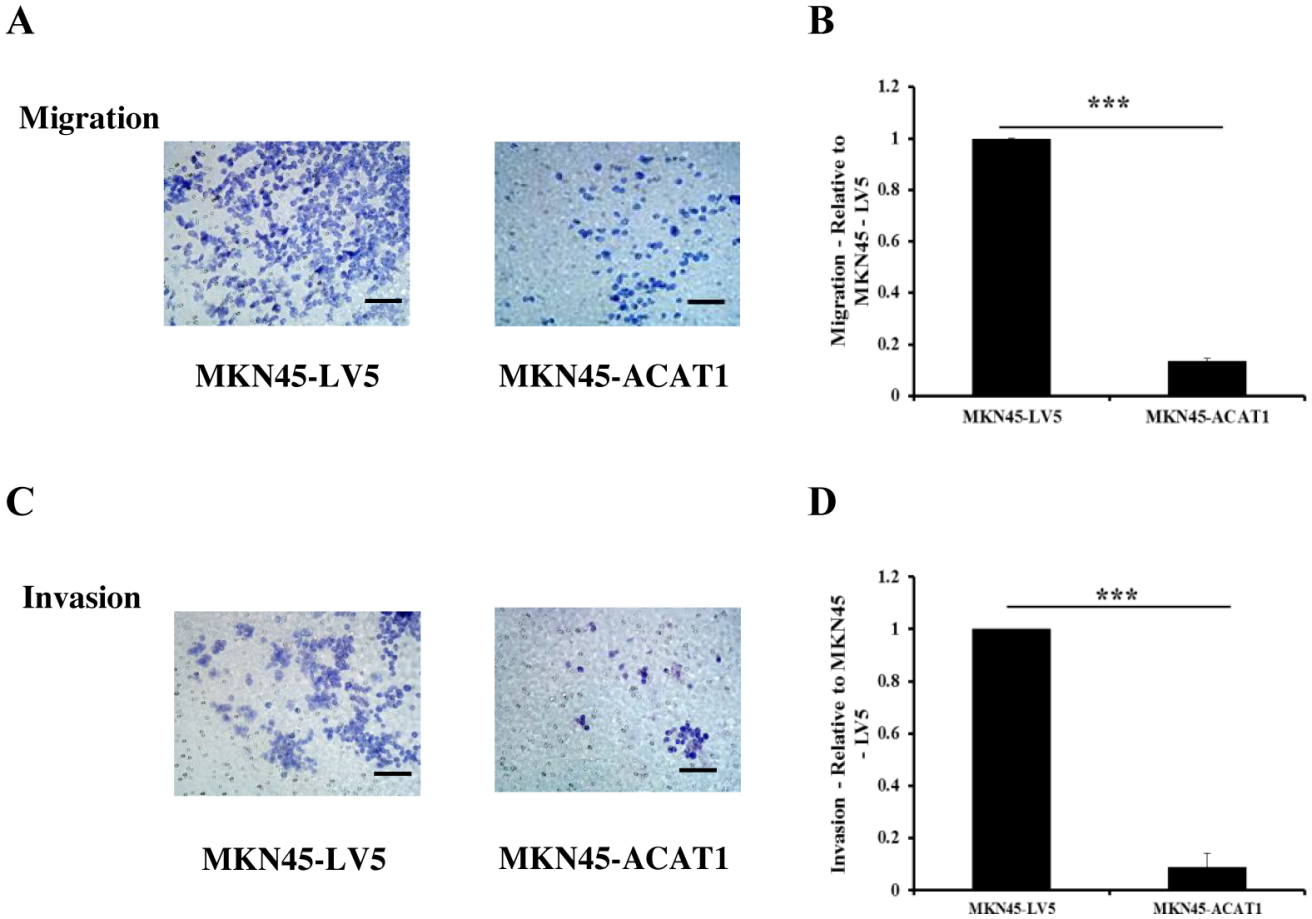
ACAT1 inhibits the migration and invasion of gastric cancer cells. **(A–D)** Transwell assay to assess the migration and invasion ability of MKN45-ACAT1 and MKN45-LV5 cells. Scale bar: 100 μm. n = 3 in each group. Values are mean ± SD. ***p < 0.001.

Tumors can enhance their invasion of nearby tissues by secreting matrix metalloproteinases (MMPs). To determine if overexpression of *ACAT1* affects the invasive potential of gastric cancer cells, we used Transwell assays. In the Transwell assay, we placed a layer of Matrigel in the chamber. Gastric cancer cells, attracted by nutrients at the bottom, secreted MMPs to pass through the Matrigel and access the nutrients needed for growth. The primary difference between cell migration and invasion assays is the presence of Matrigel, which simulates the extracellular matrix in the invasion assay. The results demonstrated that overexpression of *ACAT1* in MKN45 cells significantly reduces their migration and invasion capabilities (**Figures 5A–D**).

### ACAT1 improves the response to 5-FU, etoposide and inhibits the ability of gastric cancer cells to form colonies in soft agar

**Figure 6:**
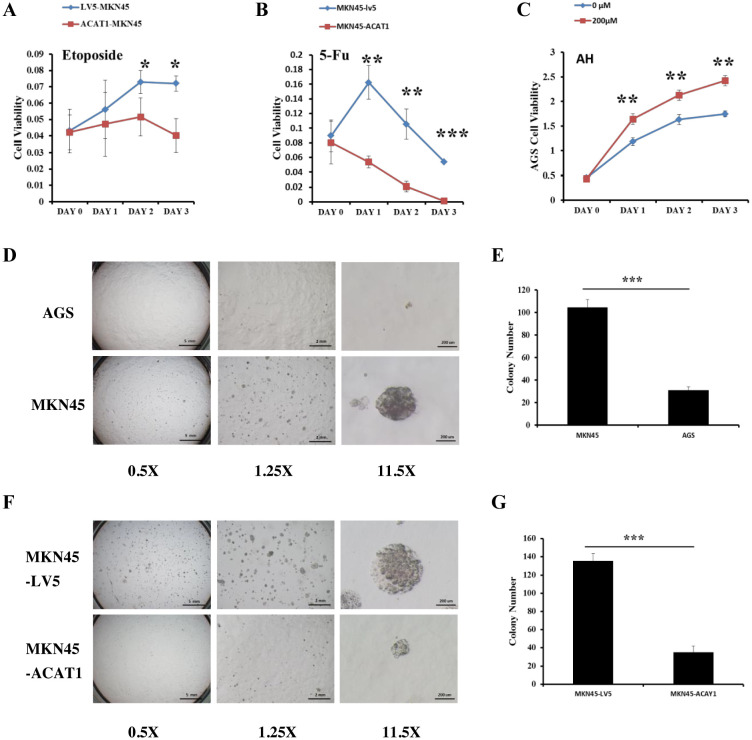
*ACAT1* overexpression improves the response to chemotherapy drugs and inhibits the ability of gastric cancer cells to form colonies in soft agar. **(A, B)** The sensitivity of MKN45-ACAT1 and MKN45-LV5 cells to the chemotherapeutic drugs 5-FU (4 mM) and etoposide (80 μM) was analyzed by CCK8 reagent. **(C)** The sensitivity of AGS cells to the arecoline hydrobromide (200 μM) was analyzed by CCK8 reagent. **(D)** The anchorage-independent proliferation of AGS and control MKN45 cells was assessed through a soft agar colony formation assay. The scale bar in the 0.5X represents 5 mm, the scale bar in the 1.25X represents 2 mm and the scale bar in the 11.5X represents 200 μm. **(E)** The number of colonies formed by AGS and MKN45 cells. **(F)** The anchorage-independent growth of MKN45-ACAT1 and MKN45-LV5 cells was detected by using a soft agar colony formation assay. The scale bar in the 0.5X represents 5 mm, the scale bar in the 1.25X represents 2 mm and the scale bar in the 11.5X represents 200 μm. **(G)** The number of colonies formed by MKN45-ACAT1 and MKN45-LV5 cells. n = 3 in each group. Values are mean ± SD. *p < 0.05, **p < 0.01, ***p < 0.001.

Chemotherapy resistance is largely due to the presence of cancer cells acquiring stemness characteristics (27). To assess whether ACAT1 affects drug response of gastric cancer stem cells, we compared the drug response of MKN45-LV5 cells and MKN45-ACAT1 cells treated with 5-Fluorouracil (5-FU) and etoposide, two commonly used chemotherapy drugs in gastric cancer. Drug response was determined by determining the number of viable cells after treatment. Our results identified a significant decrease in the survival of MKN45-ACAT1 cells compared to MKN45-LV5 cells after treatment with 5-FU or etoposide (**Figures 6A, B**).

Additionally, Fan et al. found that Arecoline Hydrobromide (AH) is an inhibitor of the ACAT1 tetramer (28). We treated AGS cells, which have high ACAT1 expression, with AH and conducted a cell proliferation assay. The study revealed that AH treatment significantly increased the growth rate of AGS cells compared to the control group (**Figure 6C**). We evaluated the ability of two gastric cancer cell lines to form colonies. The results showed that compared with AGS cells, MKN45 cells formed more and larger colonies (**Figures 6D, E**).

Thereafter, we conducted soft agar anchorage-independence colony formation assay using MKN45-LV5 and MKN45-ACAT1 cells to investigate the influence of ACAT1 on the colony-forming potential of gastric cancer cells. Compared with MKN45-LV5 cells, MKN45-ACAT1 cells exhibited a significant reduction in the number and volume of colonies developed in soft agar (**Figures 6F, G**).

### ACAT1 suppresses the growth of gastric cancer xenograft tumors in nude mice

**Figure 7:**
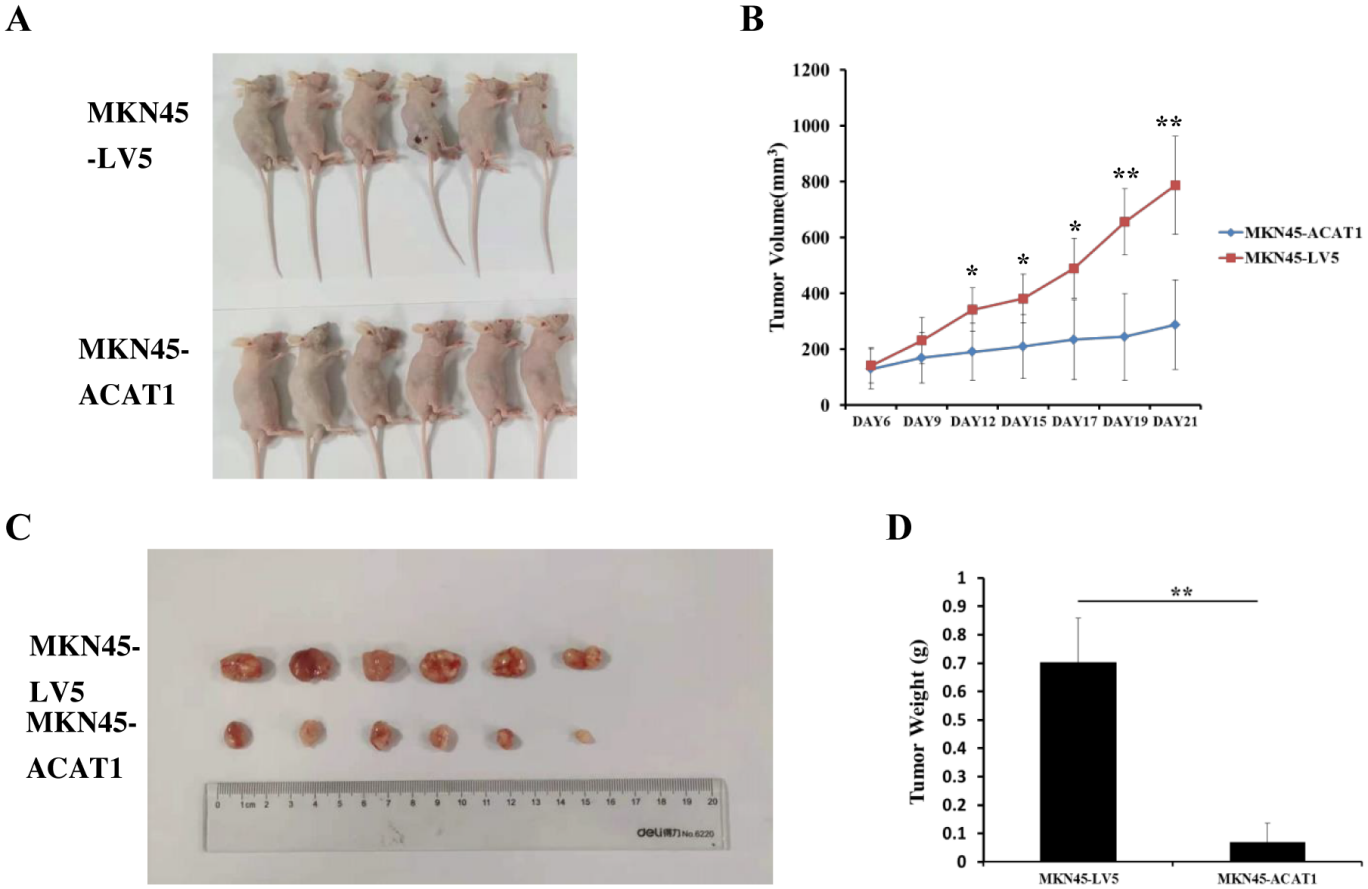
*ACAT1* overexpression inhibits tumor growth in nude mouse xenograft model. **(A)** MKN45-LV5 cells or MKN45-ACAT1 cells were injected into each nude mouse, and representative images were taken on day 21 after injection. Upper panel represents MKN45-LV5; lower panel represents MKN45-ACAT1. **(B)** The tumor volumes were measured on day 6, 9, 12, 15, 17, 19 and 21. **(C, D)** On day 21, the tumors were taken out, excised, and weighed. Upper panel represents MKN45-LV5; lower panel represents MKN45-ACAT1. n = 6 in each group. Values are mean ± SD. *p < 0.05, **p < 0.01.

We also examined the effects of increased ACAT1 expression on tumor growth *in vivo* using a xenograft mouse model. Eight million MKN45-ACAT1 or control MKN45-LV5 cells, combined with Matrigel, were injected subcutaneously into the backs of male nude mice aged 6 weeks. Tumor volumes were measured starting from day 6 post-injection. After 12 days from cell injections, there was a notable difference in tumor volume between the two cell lines groups. Compared to tumors formed by MKN45-LV5 cells, those formed by MKN45-ACAT1 cells exhibited a significant decrease in volume and inhibition of growth rate (**Figures 7A, B**). Finally, at 21 days post-injection, tumors were excised and weighed. The weight of tumors formed by MKN45-ACAT1 cells was substantially lighter than those formed by MKN45-LV5 cells (**Figures 7C, D**). These findings suggest that increased expression of ACAT1 suppresses gastric cancer tumor growth in nude mice.

### Expression of the ACAT1 gene to the expression pattern of whole genes and GSEA of the ACAT1 gene expression

**Figure 8:**
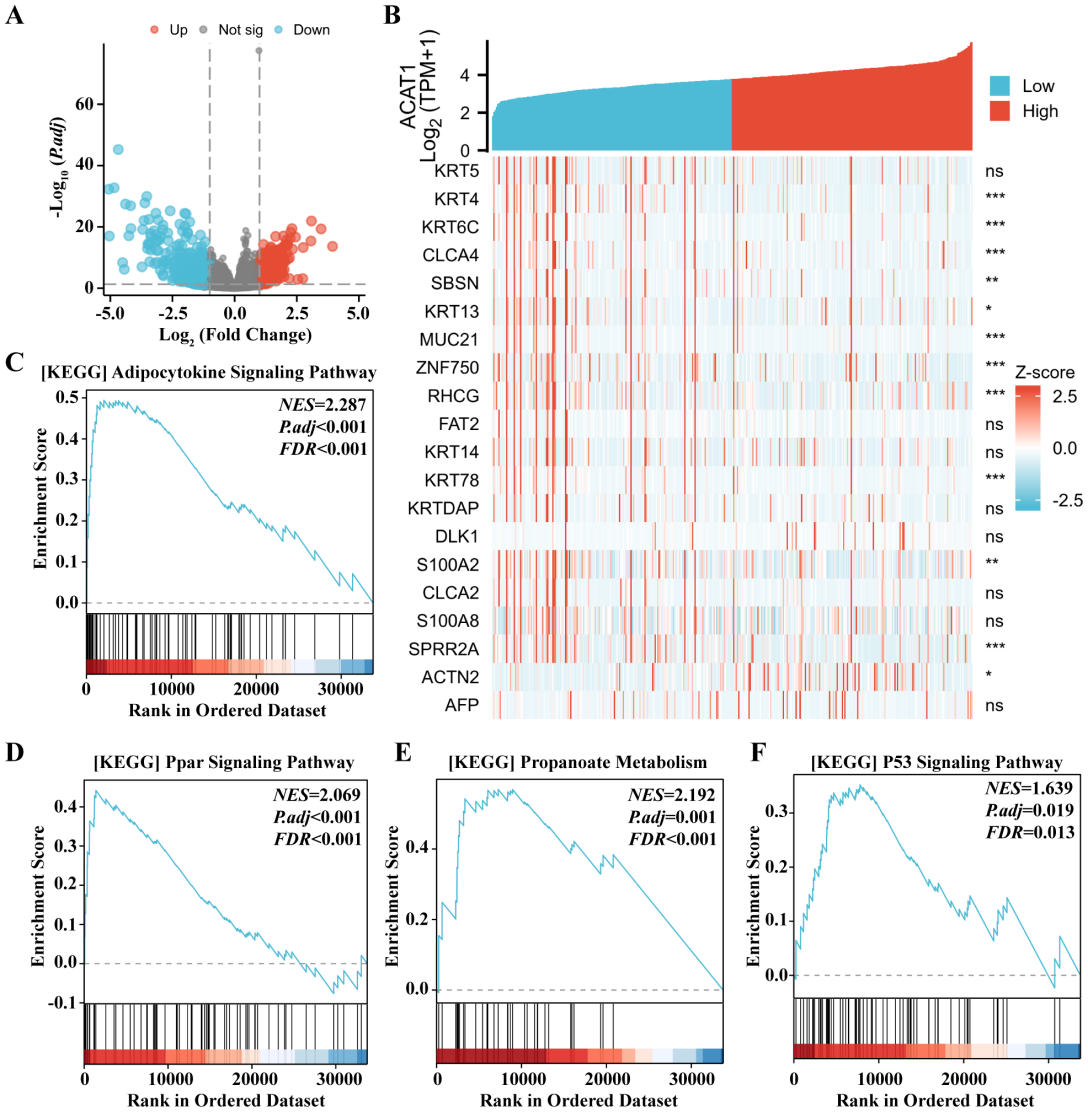
Expression of the *ACAT1* gene to the expression pattern of whole genes and bioinformatics analysis. **(A)** A volcano map based on *ACAT1* expression patterns illustrating the differentially expressed genes (DEGs). **(B)** The expression level of the *ACAT1* gene was used to generate a heat map that displays 20 genes that were either upmodulated or downmodulated. **(C–F)** The findings of the gene set enrichment analysis (GSEA). ns, not significant. *p < 0.05, **p < 0.01, ***p < 0.001.

To enhance comprehension of the biological function of the *ACAT1* gene in gastric cancer, an evaluation of the *ACAT1* gene expression pattern was conducted. It was discovered that the expression of 1822 genes that were in a downmodulated and 288 genes that were in an upmodulated were substantially linked to the *ACAT1* gene expression (logFC > 1 and *p*adj < 0.05) (**Figure 8A**). Furthermore, the gene expression heat map (**Figure 8B**) showed the top 20 genes exhibiting abnormal levels of expression (abslogFC > 2 and *p*adj < 0.01). Furthermore, an analysis was conducted using TCGA gene expression data to compare the biological and functional pathways in high- and low-*ACAT1* gene expression groups through Gene Set Enrichment Analysis (GSEA). The NESs indicated that the enrichment signaling pathway most relevant for *ACAT1* gene expression was selected. The GSEA analysis showed that the increased *ACAT1* gene expression pattern was mainly found in the Adipocytokine Signaling Pathway, Ppar Signaling Pathway, Propanoate Metabolism and P53 Signaling Pathway (**Figures 8C–F**). This suggests that the inhibitory effect of *ACAT1* in gastric cancer may be related to the above pathways.

### PPI networks and functional annotations

Through interactions with neighboring stromal cells within the tumor microenvironment (TME), tumor cells have developed adaptive mechanisms to survive under various extreme conditions such as hypoxia and elevated reactive oxygen species (ROS) (29). These stress phenotypes are considered hallmarks of cancer (30). ROS are linked to cancer initiation, development, and progression (31). To explore a potential role for the *ACAT1* in modification of the gastric cancer metabolic microenvironment, we constructed PPI networks and functional annotations based on the STRING database (https://string-db.org/), GO, and KEGG analyses. **Supplementary Figure S1A** shows a network of *ACAT1* and its 10 coexpression genes. The correlation analyses between the expression of *ACAT1* and co-expressed genes in gastric cancer from TCGA are shown in **Supplementary Figures S1B–I**. As shown in **Supplementary Figure S1J**, changes in the biological process of *ACAT1* are associated with increased intramolecular oxidoreductase activity, suggesting that *ACAT1* may suppress tumor initiation and progression by inhibiting the production of ROS.

### Correlation analysis between ACAT1 expression and immune cell infiltration in gastric cancer

**Figure 9:**
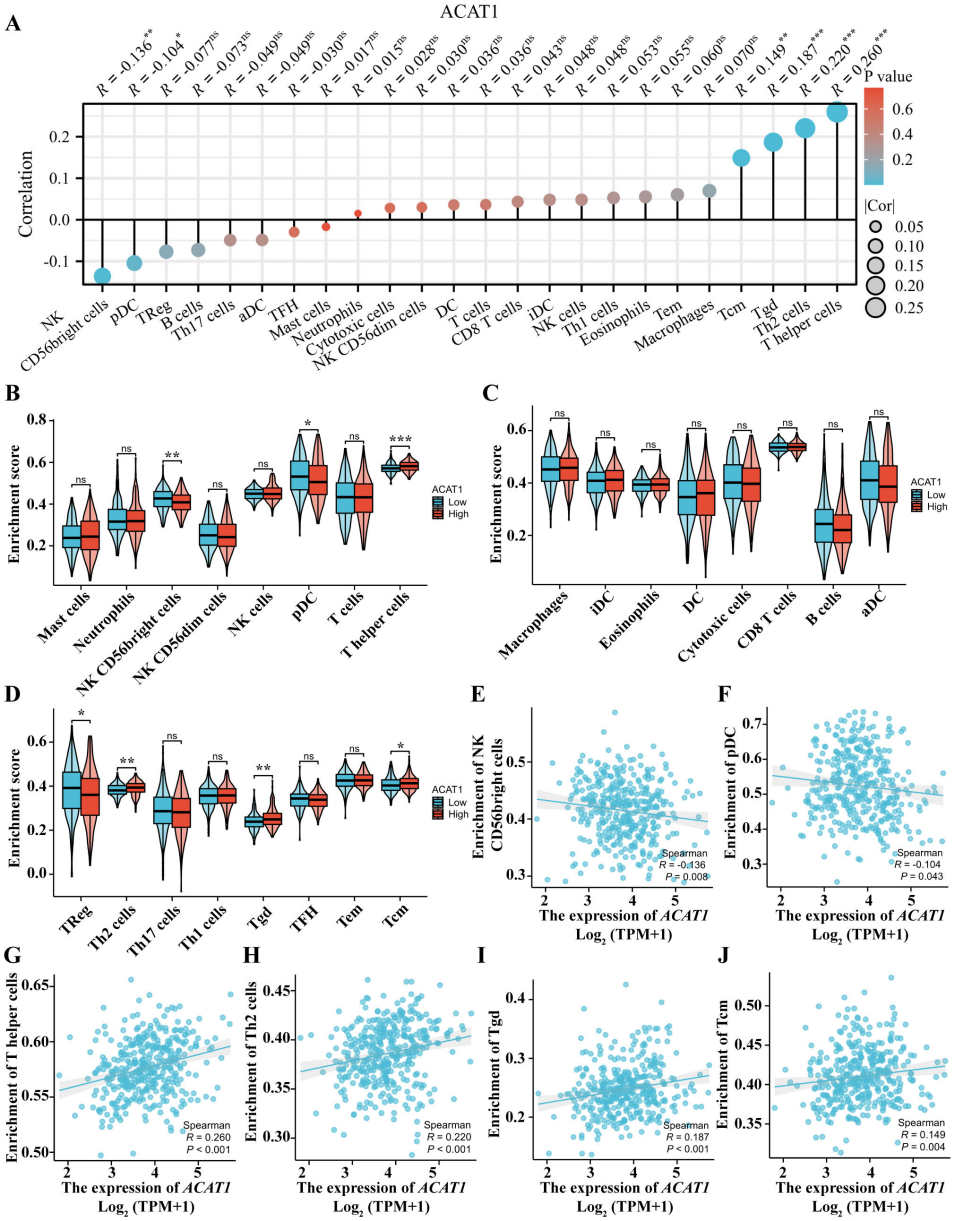
Relationship between the *ACAT1* gene expression and immune cell infiltration. **(A)** The relationship between the *ACAT1* gene expression and immune cell infiltration status. **(B–D)** Differences in the degree to which certain immune cell subsets were enriched in the *ACAT1* gene high- and low-expression groups. **(E–J)** Relationships between the *ACAT1* gene expression and tumor microenvironment characteristics. ns, not significant. *p < 0.05, **p < 0.01, ***p < 0.001.

The level of immune cell infiltration in the tumor microenvironment (TME) plays a cardinal role in the tumor initiation, progression, metastasis and treatment resistance of cancer (32). The relationship between the *ACAT1* gene expression and 24 distinct immune cell subtypes in gastric cancer was investigated and analyzed. The *ACAT1* gene expression had a strong positive correlation with T helper cells, Th2 cells, Tgd and Tcm infiltration and a strong inverse correlation with pDC and NK CD56bright cells infiltration (**Figures 9A, E–J**). Further investigation illustrated substantial variations in the *ACAT1* gene expression level in different infiltrating immune cells, notably NK CD56bright cells, pDC, T helper cells, TReg, Th2 cells, Tgd and Tcm (**Figures 9B–D**).

### Tumor samples exhibited reduced ACAT1 gene expression compared to normal samples

We examined the *ACAT1* gene expression in pan-cancer samples and compared it to the expression in adjacent healthy tissues in the cancer genome atlas (TCGA) dataset to investigate whether low *ACAT1* gene expression in cancer is a common occurrence (**Supplementary Figure S2A**). The TCGA database was used to make predictions about the patterns of *ACAT1* mRNA expression in gastric cancer and normal tissue specimens. *ACAT1* mRNA expression was notably reduced in primary tumor samples of gastric cancer compared to normal tissue samples (**Supplementary Figure S2B**). *ACAT1* expression was found to be decreased in gastric cancer tissue specimens compared to normal tissue specimens and adjoining gastric cancer tissues, based on data from GTEx (**Supplementary Figure S2C**). In addition, there was a significant decrease in *ACAT1* expression in gastric cancer samples when compared to corresponding adjoining samples (**Supplementary Figure S2D**). Furthermore, the decrease in *ACAT1* expression in gastric cancer samples compared to normal samples were confirmed using the GEO datasets (GSE27342) (**Supplementary Figure S2E**). Likewise, analysis of Human Protein Atlas (HPA) data revealed a decrease in ACAT1 protein expression in gastric cancer tissue when compared to normal tissue (**Supplementary Figure S2F**). We also analyzed the correlation of *ACAT1* expression with clinical features of gastric cancer patients through the UALCAN database. We found that lower *ACAT1* expression was correlated with higher individual stage, tumor grade, and nodal metastasis status (**Supplementary Figures S2G–I**).

### Diagnostic and prognostic relevance of ACAT1 expression in gastric cancer

A receiver operating characteristic (ROC) curve was constructed to examine the diagnostic significance of *ACAT1* expression by comparing *ACAT1* expression in normal tissue specimens (data obtained from GTEx) and adjoining gastric cancer tissues with that of gastric cancer specimens. The results showed that the area under the curve (AUC) value for *ACAT1* levels was 0.590 (confidence interval = 0.484–0.695), indicating a poor overall diagnostic accuracy (**Supplementary Figure S3A**). Besides, we analyzed the correlation of *ACAT1* expression with prognosis of gastric cancer patients from the TCGA database. The *p*-value of the overall survival (OS), disease-specific survival (DSS) and progression-free interval (PFS) curve is not significant (**Supplementary Figures S3B–D**). However, gastric cancer patients with high expression of *ACAT1* perform better than those with low expression of *ACAT1* in the PFS curve (**Supplementary Figure S3D**).

## Discussion

ACAT1, a vital mitochondrial enzyme, plays a key role in metabolic processes, including the production and degradation of ketones, as well as the breakdown of isoleucine and fatty acids (33). Reports suggest that MiR-21 targets the 3’UTR of human *ACAT1* mRNA, resulting in decreased ACAT1 levels, promoting cell growth (34). *ACAT1* is not only regulated by MiR-21 but also modulates the pyruvate dehydrogenase complex (PDC), inhibiting its activity and shifting cellular metabolism towards aerobic glycolysis.

In tissues from gastric cancer patients and in gastric cancer cell lines, we observed negative correlation between ACAT1 expression and TNM staging. Hence, we hypothesized that ACAT1 may inhibit the establishment and progression of gastric cancer. To test this hypothesis, we developed a stably transformed gastric cancer cell line to analyze cancer stem cell markers. We found that ACAT1 decreases the levels of CD44 and OCT4, increases the levels of CD24, inhibits colony formation in soft agar, and improves the response to chemotherapy drugs such as 5-FU and etoposide. The proliferation rate of AGS cells significantly increased when treated with the ACAT1 inhibitor AH. Additionally, ACAT1 suppresses the growth of gastric cancer xenograft tumors in nude mice. In summary, ACAT1 inhibits the stemness of gastric cancer cells.

In further support of tumor-suppressor role in gastric cancer, we also found that ACAT1 negatively regulates EMT. ACAT1 increases the expression of the epithelial protein E-cadherin and decreases the expression of the mesenchymal protein vimentin. Additionally, ACAT1 suppresses the transcription of *SNAI1* and *SNAI3* genes, which promote EMT. EMT is closely associated with the migratory and invasive capabilities of gastric cancer cells. Moreover, our Transwell experiments revealed that ACAT1 inhibits the migratory and invasive abilities of gastric cancer cells, indicating that ACAT1 disrupts the EMT process.

EMT involves a series of complex events through which cells transition to reduce their epithelial characteristics and become more migratory. Traditionally, this transformation was thought to be complete when cells lost their epithelial markers and gained mesenchymal markers. However, new findings suggest that cells undergoing EMT are often diverse, exhibiting varying levels of both epithelial and mesenchymal markers. Cells frequently display a partial EMT phenotype and may not require a full mesenchymal transition to migrate (35). In our study, immunofluorescence staining of MKN45-LV5 and MKN45-ACAT1 cells revealed that some cells expressed both E-cadherin and vimentin, while others expressed only E-cadherin or vimentin. Overexpression of *ACAT1* enhanced E-cadherin expression, decreased vimentin expression, and reversed the EMT process in the majority of cells. However, some cells still expressed vimentin, suggesting that these cells are in an intermediate state during EMT.

Previous studies have also shown enhanced methylation of *ACAT1* gene promoter in renal clear cell carcinoma and nasopharyngeal carcinoma, leading to decreased ACAT1 expression and a correlation with EMT initiation (36, 37). This supports our finding that downregulation of ACAT1 promotes EMT in gastric cancer.

In addition to our current results in gastric cancer, previous studies have also shown that ACAT1 expression levels in prostate cancer are higher compared to adjacent normal tissues, with *ACAT1* and *SIRT5* synergistically promoting the development of invasive prostate cancer (38). ACAT1 also sensitizes doxorubicin-resistant uterine cancer cells to doxorubicin therapy (39). In liver cancer, ACAT1 promotes lipid metabolism and tumor occurrence by stabilizing FASN through the acetylation of GNPAT (40). ACAT1 also enhances leukemia generation by acetylating mutant isocitrate dehydrogenase 2 (41). ACAT1 is dysregulated in renal cell carcinoma (42, 43), nasopharyngeal carcinoma (36), and glioma (44) compared to adjacent healthy tissues. However, there have been no reports on the involvement of ACAT1 in the progression of gastric cancer.

Zhang et al. investigated the function and mechanisms of Acetyl-CoA acetyltransferase 2 (ACAT2) in gastric cancer (45). The authors demonstrate that the expression of ACAT2 was significantly increased in GC tissues compared with normal counterparts. *In vitro* functional studies demonstrated that the proliferation and the motility of GC cells were inhibited by ACAT2 knockdown. They further showed that ACAT2 acts as an inhibitor of YAP1 ubiquitination, thereby preventing its degradation and allowing it to accumulate in cells. This accumulation enhances the proliferation and metastatic potential of gastric cancer cells. Mechanistically, the study reveals that ACAT2 achieves this by upregulating SET domain-containing protein 7 (SETD7), a lysine methyltransferase known to promote YAP1 stability. By increasing the expression of SETD7, ACAT2 facilitates the methylation of YAP1, which in turn reduces its recognition by ubiquitin ligases, thus suppressing its ubiquitination and subsequent proteasomal degradation (45).

Cancer stem-like cells (CSCs), also known as cancer-initiating cells (CICs), represent a subpopulation of cells characterized by their high tumorigenic potential, self-renewal ability, and prominent expression of stemness-specific markers such as CD133, Nanog, Sox2, and Oct4 (46). Recent studies have identified CD44 as a universal marker for CSCs/CICs (47). In colon cancer, CD44(hi) cancer stem cells demonstrate an enhanced ability to form colonies in soft agar (48). Remarkably, as few as 10 CD44(hi) cells were capable of forming tumors in 70% of mice, whereas CD44(-) colon tumor cells were non-tumorigenic. Furthermore, CD44(hi) cells could be serially passaged *in vivo*, indicating their self-renewal capacity, and they formed tumors that recapitulated the heterogeneity of the original patient tumor. Bromodeoxyuridine (BrdU) labeling studies have shown that CD44(hi) cells divide more slowly than CD44(-) cells, suggesting that their tumorigenicity is not merely due to faster proliferation (48). These CSCs have been identified in several human solid tumors, including gastric cancer (2–6, 9–12). CSCs exhibit resistance to conventional chemotherapy and radiotherapy, contributing to tumor relapse and metastasis (49). Another key characteristic of CSCs is their involvement in epithelial-mesenchymal transition (EMT). Inhibiting EMT transcription factors such as TWIST, SNAIL, and SLUG, or targeting GSK-3β, has been proposed as a strategy to block the stemness properties of CSCs (50). We demonstrated that overexpression of *ACAT1* reduces levels of CD44 and OCT4, inhibits colony formation in soft agar, enhances the response to chemotherapy drugs, and negatively regulates EMT. Our data suggest that ACAT1 acts as an inhibitor of cancer initiation.

In summary, we found that the expression of ACAT1 is downregulated in advanced gastric cancer. ACAT1 inhibits the migration and invasion, EMT process, and survival of gastric cancer cells. In bioinformatic analyses, ACAT1’s inhibitory effect on gastric cancer may be related to the Adipocytokine Signaling Pathway, the Ppar Signaling Pathway, propanoate metabolism, and the P53 Signaling Pathway. Immune infiltrates were correlated with the expression of *ACAT1* mRNA. A limitation of our study is the lack of a deeper mechanistic understanding the ACAT1’ s role in the stemness and EMT of gastric cancer cells. Further research is needed to evaluate the functional role of ACAT1 in gastric cancer.

## Materials and methods

### Patients and tumor tissues

To investigate the role of ACAT1 in gastric cancer, we collected cancer tissues and adjacent tissues of gastric cancer patients at different stages from the First Affiliated Hospital of Suzhou University in Jiangsu Province, China. This study involved 88 patients, all diagnosed with gastric cancer through histopathology, and underwent resection of gastric cancer between August 2019 and May 2020. None of the patients received anticancer therapy before surgery. The tumors were staged according to the 2010 WHO Digestive System Tumor Classification Standard. Collected samples were immediately frozen in liquid nitrogen and stored for subsequent analysis.

### Animals and gastric cancer xenograft tumors

Male BALB/c-Nude mice (6-8 weeks old males, SPF grade) were procured from Jiangsu Jicui Yaokang Biological Co., Ltd. Mice were housed in an SPF animal facility at Soochow University. They were kept in individually ventilated cages (IVC), Euro Standard Type IIL in groups of six with filter tops, and were provided with drinking water and standard chow ad libitum. The temperature in animal facilities was 22–26 °C, and the humidity was 55 ± 10%.

### Cell lines and tissues

The human gastric cancer cell line MKN45 (Beijing Institute of Cell Biology, Chinese Academy of Sciences) and the human gastric cancer cell line N87 (Shanghai Institute of Cell Biology, Chinese Academy of Sciences) were cultured in RPMI 1640 medium (1% PS and 10% FBS). The human gastric cancer cell line AGS (Shanghai Institute of Cell Biology, Chinese Academy of Sciences) was cultured in F-12K medium (1% PS and 10% FBS). The human renal epithelial cells 293T (Shanghai Institute of Cell Biology, Chinese Academy of Sciences) was cultured in DMEM medium (1% PS and 10% FBS).

### Antibodies

ACAT1 (TA392867, 1:1000 dilution for WB, 1:100 dilution for IF) was from Origene. GAPDH (2118S, 1:5000 dilution for WB), E-cadherin (14472S, 1:1000 dilution for WB, 1:100 dilution for IF), Vimentin (5741S, 1:1000 dilution for WB, 1:100 dilution for IF), CD44 (3570S, 1:400 dilution for IF), Anti-mouse IgG (HRP-linked, 7076S, 1:5000 dilution for WB), and Anti-rabbit IgG (HRP-linked, 7074S, 1:5000 dilution for WB) were from Cell Signaling Technology. CD44-PE (550989, 1:50 dilution for FCM) and CD24-PerCP-Cy5.5 (561647, 1:50 dilution for FCM) were from BD. CD24 (ab202073, 1:100 dilution for IF) was from Abcam. Alexa Fluor 488 goat anti-mouse IgG1 (A21121, 1:500 dilution for IF), Alexa Fluor 488 goat anti-mouse IgG(H+L) (A11001, 1:500 dilution for IF), Alexa Fluor 568 goat anti-rabbit IgG(H+L) (A11010, 1:500 dilution for IF), Alexa Fluor 568 goat anti-mouse IgG2a (A21134, 1:500 dilution for IF) were from Thermo Fisher Scientific.

### Western blot

Cell or tissue samples were treated with lysis buffer from Cell Signaling Technology (46232S) and centrifuged to remove insoluble particles. Proteins were separated using SDS-PAGE and subsequently transferred to a PVDF membrane. The membrane was blocked with 5% nonfat milk powder for 2 hours at room temperature. Primary antibodies were incubated with the membranes overnight at 4°C. After three washes with TBST solution, membranes were exposed to secondary antibodies. The bands were visualized using ECL chemiluminescence reagent (PerkinElmer, NEL120001EA).

### Immunofluorescence staining

When the cells reached a density of 80% - 90%, the initial medium was removed, and the cells were rinsed with PBS. They were then treated with immunostaining fixative solution (Beyotime, P0098) for 10 minutes at room temperature. Following this, the cells were washed and treated with Triton X-100 solution (Sigma-Aldrich, 9002-93-1) for 10 minutes at room temperature to permeabilize them. After blocking with immunostaining blocking solution (Beyotime, P0102), primary antibodies were added and incubated with the cells overnight at 4°C. The next day, secondary antibodies were applied for 1 hour at room temperature in the dark. Finally, the cells were incubated with DAPI (Sigma-Aldrich, D8417) diluted in distilled water for 5 minutes at room temperature.

### RT-qPCR

RNA was extracted using the Trizol solution (Invitrogen, 15596026). Primers for RT-qPCR were created using the Origene website (https://www.origene.com/) and the NCBI-Primer BLAST website (https://www.ncbi.nlm.nih.gov/) for validation. Hongxun Biological Co., Ltd. synthesized the primers. The Ct value was measured using the Quant Studio 6 real-time fluorescence quantitative PCR instrument, and the relative expression level of mRNA was determined using the 2^-ΔΔCt^ method. The RT-qPCR primer set sequences are shown as **Supplementary Table S1**.

### Construction of ACAT1 overexpressing cells

The *ACAT1* cDNA was amplified using primers that containing the SphI and BamHI restriction site sequence, utilizing the KOD enzyme reaction system from Toyobo (KOD201). The item was replicated in the LV5 plasmid from GenePharma. Lentivirus produced in 293T cells was used to infect the gastric cancer cells, which were then treated with Puromycin for screening (51). The sequences of *ACAT1*-CDS and *ACAT1*-overexpression primers are shown as **Supplementary Table S2**.

### Flow cytometric analysis

We used 1 x 10^7^ gastric cancer cells for flow cytometry analysis. Following incubation with the initial antibodies, the cells were washed and then passed through a 70 μm filter before being examined using a flow cytometer.

### Migration and invasion assays

The procedures for migration and invasion have been described in detail in our previous publication (52). The procedure for cell migration is similar to that of invasion, except that Matrigel was not added.

### Drug sensitivity experiments

Three thousand cells were resuspended in 200 μl of medium (either normal or containing drugs) and placed in a 96-well plate. The plate was then incubated at 37°C with 5% CO_2_ for 30 minutes. After incubation, 20 μl of CCK8 reagent was added to each well at day 0, 1, 2 and 3. The optical density (OD) at 450 nm was measured using a microplate reader after incubation with CCK8 reagent for 2 h. The OD value of the blank medium served as a control, and the relative levels of proliferation were determined after standardization.

### Colony formation in soft agar

A soft agar assay was conducted in a Petri dish containing 3000 cells following the procedure we have previously described with modifications (53). The bottom layer contains 0.5% agarose and the top layer contains 0.4% low melting point agarose.

### Data source

The TCGA, a data platform accessible at https://portal.gdc.cancer.gov, offers clinicopathological information on 33 cancer types as part of a major genome project, easily accessible to researchers and scholars. Clinical information of patients with gastric cancer and high-throughput RNA sequencing (RNA-seq) data was retrieved from the TCGA database. The Human Protein Atlas (HPA) provides comprehensive information on the gene expression and protein profiles of diverse human samples, such as tissues, cells, and disease states. At present, the online database includes information about the unique locations of 44 healthy tissues and the top twenty commonly identified types of cancer. Additionally, the database includes information on protein immunohistochemistry in both tumor and normal human tissue samples.

### GEO analysis

All microarray data was downloaded from the GEO database (http://www.ncbi.nih.gov/geo). Box plots are drawn by boxplot of R.

### UALCAN analysis

The UALCAN website offers a comprehensive and interactive analysis of bioinformatics using RNA-seq and clinical information from 31 different types of cancers in TCGA database (http://ualcan.path.uab.edu/). The database has the capability to analyze gene expression across various tumor stages or subtypes, along with additional clinicopathological characteristics. We analyzed the level of *ACAT1* expression in relation to key clinical characteristics including cancer stage, grade of tumor, and presence of nodal metastasis.

### GSEA analysis

The GSEA method (54) was utilized in every study to rank the genome a thousand times and identify pathways linked to *ACAT1* gene expression. During the GSEA examination, it was established that the critical value for significant results was an adjusted *p*-value less than 0.05 and a false discovery rate (FDR) below 0.25. Enrichment analysis results were determined based on the normalized enrichment scores (NESs) and adjusted *p*-values.

### Analysis of the infiltration of immune cells

The analysis of the infiltration of immune cells were performed using a method as described (55).

### Clinical statistical analysis of diagnosis and prognosis

This part was performed as previously described (55).

### Statistical methods

All experiments were repeated more than 3 times, and the data were processed by Excel, GraphPad Prism, and SPSS Statistics. The data were expressed as means ± SD, and statistical significance was analyzed by t test. Western blot was used to detect the data of gastric cancer patient tissue samples by nonparametric test Mann-Whitney analysis. n=3 in all experiments except the nude mouse tumor formation experiment, where n=6. In all results, *p* < 0.05 indicates that the data difference is statistically significant.

## Data Availability

The original contributions presented in the study are included in the article/**Supplementary Material**. Further inquiries can be directed to the corresponding authors.
