# Supplementary material for: From mitochondria to tumor suppression: ACAT1's crucial role in gastric cancer
Source: Front Immunol. 2024 Aug 23;15:1449525. doi: 10.3389/fimmu.2024.1449525 (PMC11377227; doi:10.3389/fimmu.2024.1449525)
Supplement: Supplementary file 1 [file Image1.pdf]

Supporting Information for

From Mitochondria to Tumor Suppression: ACAT1's Crucial Role in Gastric Cancer

Wei He[1#], Yanfang Li[1#], Song-Bai Liu[2*#], Ying Chang[1#], Shiyuan Han[1], Xingyu Han[1], Zixin Ma[1], Hesham M Amin[3], Yao-Hua Song[1*], Jin Zhou[4*]

[1]Cyrus Tang Hematology Center, Soochow University, Suzhou, 215123, P.R. China

[2]Suzhou Key laboratory of medical biotechnology, Suzhou vocational health college, Kehua Road 28, Suzhou, P.R. China 215009

[3]Department of Hematopathology, the University of Texas MD Anderson Cancer Center, 1515 Holcombe Boulevard, Houston, Texas, USA

[4]Department of General Surgery, the First Affiliated Hospital of Soochow University, Suzhou, P. R. China

[#]These authors contributed equally to this work.

***Corresponding author:** Jin Zhou/ Song-Bai Liu/ Yao-Hua Song

Email: 13913506369@163.com/ liusongbai@126.com/ yaohua_song1@yahoo.com

This file includes:

Figures S1 to S3

Supplementary Figure 1

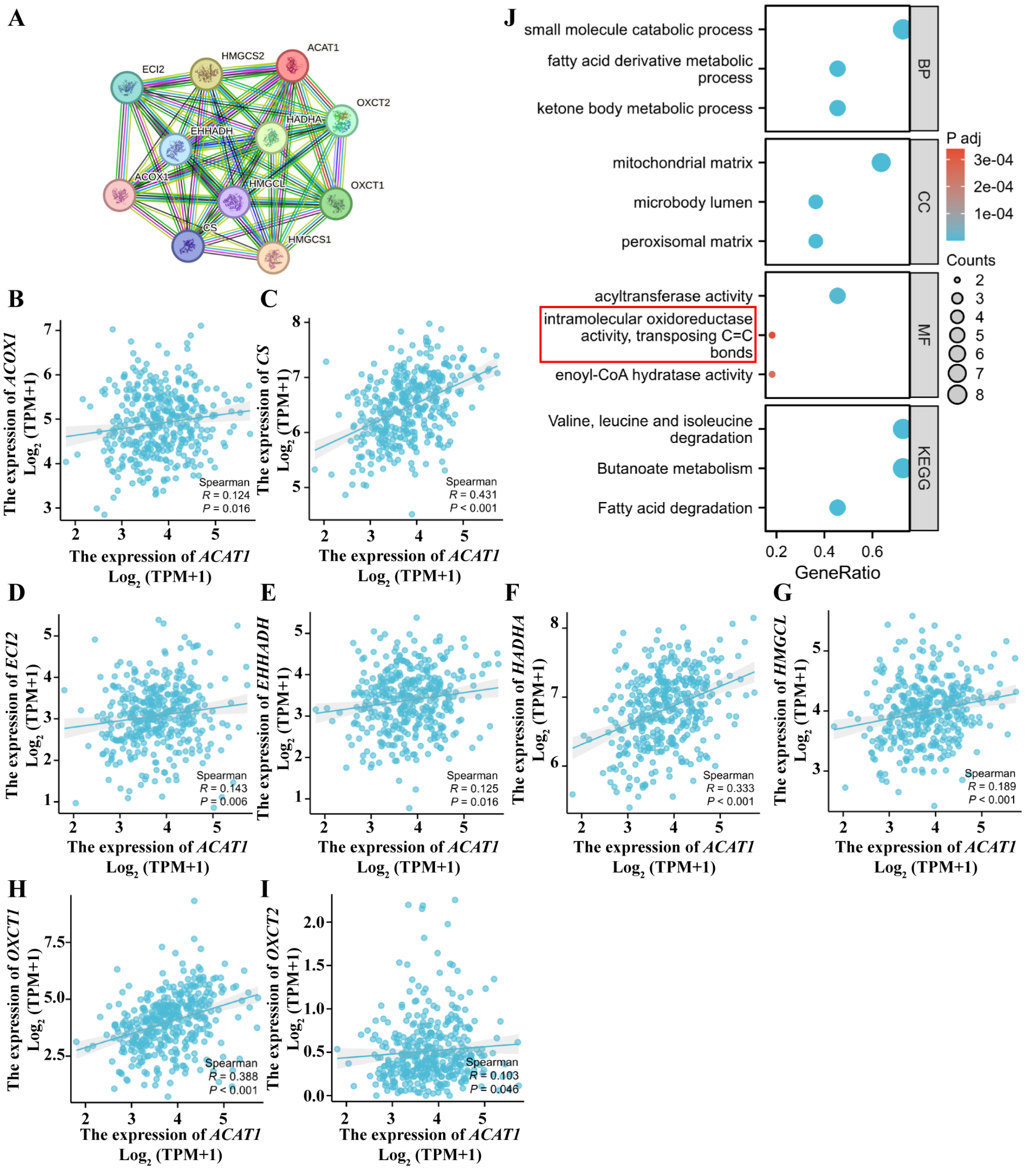

Supplementary Figure 1. PPI networks and functional enrichment analyses. (A) A network of *ACAT1* and its co-expression genes. (B–I) The correlation analyses between the expression of *ACAT1* and co-expressed genes in gastric cancer. (J) Functional enrichment analyses of 10 involved genes.

Supplementary Figure 2

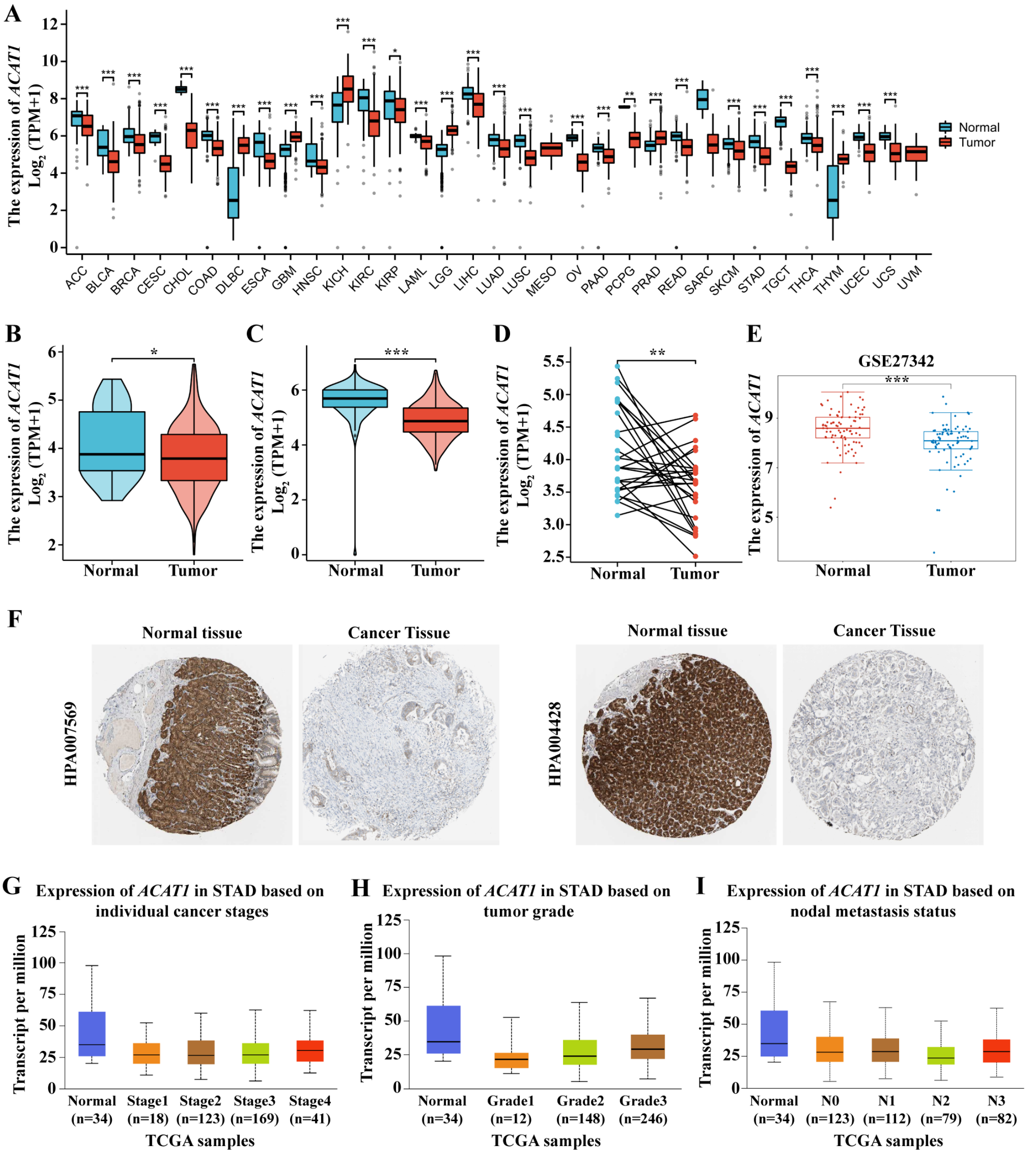

Supplementary Figure 2. Status of *ACAT1* expression in malignancies. (A) Profile of *ACAT1* expression in distinct human tumors and homologous healthy tissues. ACC, Adrenocortical carcinoma; BLCA, bladder urothelial carcinoma; BRCA, breast invasive carcinoma; CESC, Cervical squamous cell carcinoma and endocervical adenocarcinoma; CHOL, cholangiocarcinoma; COAD, colon adenocarcinoma; DLBC, Lymphoid Neoplasm Diffuse Large B-cell Lymphoma; ESCA, esophageal carcinoma; GBM, glioblastoma mutiforme; HNSC, head and neck squamous cell carcinoma; KICH, kidney chromophobe; KIRC, kidney renal clear cell carcinoma; KIRP, kidney renal papillary cell carcinoma; LAML, Acute Myeloid Leukemia; LGG, Brain Lower Grade Glioma; LIHC, liver hepatocellular carcinoma; LUAD, lung adenocarcinoma; LUSC, lung squamous cell carcinoma; MESO, Mesothelioma; OV, Ovarian serous cystadenocarcinoma; PAAD, Pancreatic adenocarcinoma; PCPG, Pheochromocytoma and Paraganglioma; PRAD, prostate adenocarcinoma; READ, rectum

adenocarcinoma; SARC, Sarcoma; SKCM, Skin Cutaneous Melanoma; STAD, stomach adenocarcinoma; TGCT, Testicular Germ Cell Tumors; THCA, thyroid carcinoma; THYM, Thymoma; UCEC, uterine corpus endometrial carcinoma; UCS, Uterine Carcinosarcoma; UVM, Uveal Melanoma. (B) Differences in *ACAT1* expression between gastric cancer tissues and adjacent healthy tissues. (C) Differences in *ACAT1* expression between normal samples (obtained using GTEx data) and adjoining gastric cancer tissues and samples. (D) Differences in *ACAT1* expression between gastric cancer samples and corresponding adjoining samples. (E) Verification of the decreased *ACAT1* expression in gastric cancer compared to normal tissues in the GSE27342 dataset. (F) ACAT1 protein expression in gastric cancer tissue was lower than in normal tissue in the HPA data (Antibody HPA007569, HPA004428, and 10X). (G-I) Association between *ACAT1* expression and clinical-pathological parameters of gastric cancer. The association between *ACAT1* expression and individual cancer stages (G), tumor grade (H) and nodal metastasis status (I). p (Normal vs Stage1, Normal vs Stage2, Normal vs Stage3, Normal vs Grade 1, Normal vs Grade 2, Normal vs Grade 3, Grade 1 vs Grade 3, Grade 2 vs Grade 3, Normal vs N0, Normal vs N1, Normal vs N2, Normal vs N3) < 0.001.Values are mean ± SD. *$p < 0.05$, **$p < 0.01$, ***$p < 0.001$.

Supplementary Figure 3

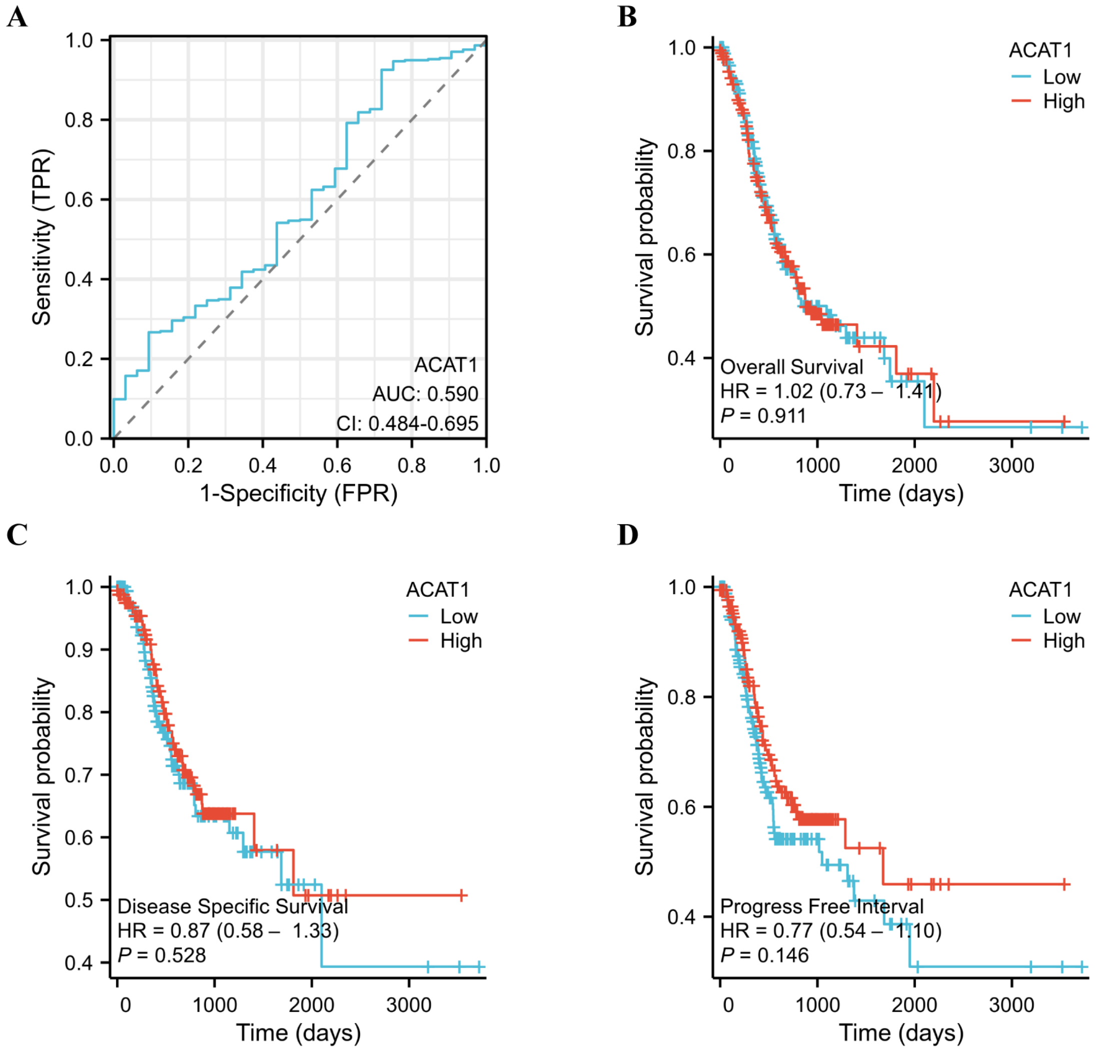

Supplementary Figure 3. Diagnostic and prognostic relevance of *ACAT1* expression in gastric cancer. (A) Receiver operating characteristic curve for *ACAT1* expression in normal samples (obtained using GTEx data) and adjoining gastric cancer tissues and samples. (B-D) The prognostic value of *ACAT1* in overall survival (OS), disease-specific survival (DSS) and progression-free interval (PFS).
