# Supplementary material for: From mitochondria to tumor suppression: ACAT1's crucial role in gastric cancer
Source: Front Immunol. 2024 Aug 23;15:1449525. doi: 10.3389/fimmu.2024.1449525 (PMC11377227; doi:10.3389/fimmu.2024.1449525)
Supplement: Supplementary file 2 [file Table1.docx]

**Supplemental Table 1: Sequences of RT-qPCR primers.**

| Gene | Forward | Reverse |
| --- | --- | --- |
| *ACAT1* | CCAGCCACTAAGCTTGGTTCCA | GTAGGAGCTTGTCCTTCACCTC |
| *CD44* | CTGCCGCTTTGCAGGTGTA | CATTGTGGGCAAGGTGCTATT |
| *OCT4* | CTTGAATCCCGAATGGAAAGGG | GTGTATATCCCAGGGTGATCCTC |
| *GAPDH* | GTCTCCTCTGACTTCAACAGCG | ACCACCCTGTTGCTGTAGCCAA |

**Supplemental Table 2: The CDS sequences of *ACAT1*.**

| Gene | Sequence |
| --- | --- |
| *ACAT1-*CDS | ATGGCTGTGCTGGCGGCACTTCTGCGCAGCGGCGCCCGCAGCCGCAGCCCCCTGCTCCGGAGGCTGGTGCAGGAAATAAGATATGTGGAACGGAGTTATGTATCAAAACCCACTTTGAAGGAAGTGGTCATAGTAAGTGCTACAAGAACACCCATTGGATCTTTTTTAGGCAGCCTTTCCTTGCTGCCAGCCACTAAGCTTGGTTCCATTGCAATTCAGGGAGCCATTGAAAAGGCAGGGATTCCAAAAGAAGAAGTGAAAGAAGCATACATGGGTAATGTTCTACAAGGAGGTGAAGGACAAGCTCCTACAAGGCAGGCAGTATTGGGTGCAGGCTTACCTATTTCTACTCCATGTACCACCATAAACAAAGTTTGTGCTTCAGGAATGAAAGCCATCATGATGGCCTCTCAAAGTCTTATGTGTGGACATCAGGATGTGATGGTGGCAGGTGGGATGGAGAGCATGTCCAATGTTCCATATGTAATGAACAGAGGATCAACACCATATGGTGGGGTAAAGCTTGAAGATTTGATTGTAAAAGACGGGCTAACTGATGTCTACAATAAAATTCATATGGGCAGCTGTGCTGAGAATACAGCAAAGAAGCTGAATATTGCACGAAATGAACAGGACGCTTATGCTATTAATTCTTATACCAGAAGTAAAGCAGCATGGGAAGCTGGGAAATTTGGAAATGAAGTTATTCCTGTCACAGTTACAGTAAAAGGTCAACCAGATGTAGTGGTGAAAGAAGATGAAGAATATAAACGTGTTGATTTTAGCAAAGTTCCAAAGCTGAAGACAGTTTTCCAGAAAGAAAATGGCACAGTAACAGCTGCCAATGCCAGTACACTGAATGATGGAGCAGCTGCTCTGGTTCTCATGACGGCAGATGCAGCGAAGAGGCTCAATGTTACACCACTGGCAAGAATAGTAGCATTTGCTGACGCTGCTGTAGAACCTATTGATTTTCCAATTGCTCCTGTATATGCTGCATCTATGGTTCTTAAAGATGTGGGATTGAAAAAAGAAGATATTGCAATGTGGGAAGTAAATGAAGCCTTTAGTCTGGTTGTACTAGCAAACATTAAAATGTTGGAGATTGATCCCCAAAAAGTGAATATCAATGGAGGAGCTGTTTCTCTGGGACATCCAATTGGGATGTCTGGAGCCAGGATTGTTGGTCATTTGACTCATGCCTTGAAGCAAGGAGAATACGGTCTTGCCAGTATTTGCAATGGAGGAGGAGGTGCTTCTGCCATGCTAATTCAGAAGCTGTAG |
| Overexpression-*ACAT1*-F | CATGCATGCATGGATTACAAGGATGACGACGATAAGGCTGTGCTGGCGGCACTT |
| Overexpression-*ACAT1*-R | CGCGGATCCCTACAGCTTCTGAATTAGCATGGCAG |
